# Port state control at European Union under pandemic outbreak

**DOI:** 10.1186/s12544-020-00460-4

**Published:** 2020-12-20

**Authors:** Efe Akyurek, Pelin Bolat

**Affiliations:** Istanbul Technical University, Maritime Faculty, Istanbul, Turkey

**Keywords:** Port state control, Pandemic outbreak, Maritime regulations, Ship inspection, Deficiency-detention factors

## Abstract

**Introduction:**

Port State Control (PSC) is a vital element of sustainable maritime transportation. Inspections of PSC regimes have been ensuring the continuity of the global supply chain as they enforce shipping to implement the maritime regulations to be safe, secure, and environmentally friendly.

**Objective:**

Since the beginning of the COVID-19 Pandemic, the number of onboard ship inspections has decreased inherently for protecting PSC officersand seafarers, while PSC regimes have developed a policy to focus on a high-risk ship based on historical inspection records. In this context, planning for a “new normal” in maritime transportation, it is essential to realize the change in ship inspection numbers and the trend of deficiency - detention factors for the maritime sector to provide current standards. This study aims to present the difference in ship inspection trends between 2017-2020 by focusing on COVID-19 pandemic outbreak data.

**Methods:**

Comparative analysis with Paris MOU ship inspection & detention figures and entropy-based Grey Relevance Analysis has been used as a methodology to reveal the change in inspection trends after COVID-19.

**Results:**

After the pandemic outbreak caused by COVID-19, the number of ship inspections under Paris MOU fell dramatically, however, inspection and detention rate remained the same, also entropy-based Grey Relevance Analysis indicates that detention remarks have also changed compared to last year deficiencies. Detention caused by nautical publication and cleanliness in the engine room has an increasing trend on detentions.

**Conclusion:**

Inspection statistics indicate consistent figures even during the pandemic outbreak, which indicates the current sample group for Paris MOU inspection is healthy. At the same time, entropy-based Grey Relevance Analysis presents a broader insight that the inspection trend on detention deficiencies has varied. Familiarization with the changing trends in inspections will cause fewer detentions of the ships.

**Supplementary Information:**

The online version contains supplementary material available at 10.1186/s12544-020-00460-4.

## Introduction

Lessons learned from the accidents present that international maritime standards are needed for the protection of human life at sea and pollution prevention of ships. Since the pandemic outbreak is a major threat to human life, it becomes subject to maritime regulations. At this point, the industry required guidelines to raise awareness against COVID-19 and calm down interested parties such as ship crew, managers, owners, inspectors, and policymakers.

The pandemic outbreak is not a new issue for maritime, especially the cruise industry. The industry even had clues of Pandemic by identifying H1N1 on a cruise ship in 2009 [32]. However, the impacts of H1N1 on mortality rate apparently remain lower than COVID-19 [30]. It appeared that H1N1 had high transmissibility but did not cause a pandemic due to it not being as severe as COVID-19 [29].

On the 5th of February, vessel Diamond Princess^1^ had an outbreak of an infectious virus known as SARS-CoV-2. Only fifteen days after the first person with COVID-19 disease, on the 20th of February, 634 persons onboard were also infected [16]. Diamond Princess was the first known case for the maritime aspect of the COVID-19 outbreaks. However, lack of regulation and guidelines on the pandemic outbreak led the maritime industry into chaos, starting with cruise ships under the effects of COVID-19. Local port authorities, shipping companies, ship sanitation inspectors had a lack of awareness against COVID-19. Even before COVID-19, hygiene inspection on ship accommodation was challenging because of the requirement of vast knowledge and experience for this complex inspection procedure [19]. Even though the majority of the shipping companies also have taken several precautions against the pandemic outbreak, such as supplying more disinfectant, gloves, and face masks, anybody coming onboard brought a risk of infection for all crew.

The maritime regulations have to be ‘proactive’ [25], but the shipping industry has not been prepared for any pandemic outbreak due to the multi-nationality of the crew and the international nature of the industry. Ships have always been a compelling factor for contagious diseases because ships are traveling long distances [3]. However, at this point, a further question arises on what deficiency caused detention during this outbreak and based on detention remarks and if there is any significant change in the Port State Control Regimes attitude or professional judgement of PSC officers in the European Union. Accordingly, this paper aims to understand the change in ship inspection numbers and evaluate the trend of ship deficiency and detention factors for the European Union due to the COVID-19 Pandemic Outbreak.

With this aim, this paper studies inspection data from Paris Memorandum^2^ Understanding (Paris MOU) [11] because all ship inspection data for European Union presented along with the detailed information and deficiency codes. As Paris MOU is a PSC regime under the European Maritime Safety Agency (European Union Port State Control Directive 2009/16/EC) has been established for European Union to support the goals of the International Maritime Organization by inspecting the regulations [20].

The paper introduces itself with four sections. Firstly, *Background* part explains the role of IMO and international regulations, Port State Control, Paris MOU, and emerging COVID-19 regulations as well. Analysis (*Statistics of Paris MOU and Grey Analysis during COVID-19 outbreak*), *Results and Discussions*, and *Conclusion*. Inspection data has been evaluated with two different approaches in the following part. In the Analysis, first of all, a comparative analysis over the last five years (2015–2020) gives a better understanding of the alteration in ship inspection numbers to see the change due to COVID-19 Pandemic. Secondly, based on the findings in the statistical analysis, the following analysis focused on 2017–2020. The inspection results and related deficiency/detention factors have been assessed with Entropy-based Grey Relational Analysis for the six months of each year between 2017 and 2020 to evaluate the variations in PSC results with the COVID-19 Pandemic. Further on, key results have been discussed, and lastly, a brief conclusion has been given.

## Background

The implementation of international maritime regulations has revolutionized the maritime industry by enforcing a safer, cleaner, and more convenient environment [23]. Indeed, the majority of the international regulations are based on the International Maritime Organization (IMO) as a special agency of the United Nations (UN) [12]. The International Maritime Organization aims to bring safety, security, environmentally green, and energy efficiency. Therefore, the key to success depends on a better implementation of these regulations that have been emphasized by IMO [23]. Although the responsibility of implementation of shipping regulations has been on shipping lines, ship owners/ managers, ship crew, and flag state where ship registered, better implementation of maritime regulations has been directly related to Port State Control (PSC) [18].

PSC can be defined as a mechanism by standing on seven international conventions.^3^ It monitors and controls the compliance of international maritime regulations of shipping for maintaining a sustainable maritime transportation system. Accordingly, it can be concluded that the primary function of port state control is to inspect compliance with international maritime regulations on merchant ships at the port of call [9]. Port State Control is a very effective tool in reducing the number of substandard ships as well as improving maritime safety and pollution prevention [1]. In recent years, there has been a significant increase in PSC activity worldwide in concert with a number of amendments to relevant international conventions.

The process of PSC has been conducted by PSC Officers (PSCO), who carry out the inspection at the ship’s port of call. The nature of the inspection requires an in-person attendance to the vessel. Thus, all inspections are done by maritime experts (masters, chief engineers and/or naval engineers), who have been trained to become an inspector by governmental administrations [6]. Port State Control is a kind of inspection that requires an onboard attendance to observe the subject vessel’s actual condition and compliance with International Maritime Regulations. Followingly, the Port State Control inspection is carried out under a marine expert with professional judgement. In case a Port State Control Officer raises a deficiency, it should be categorized to keep inspection standards. However, a major breach of international maritime regulations caused the detention of the ship by Port State Control. Detention number is also an essential criterion that indicates how many ships are unable to comply with International Maritime Regulations onboard. In relation to the inspection number, the detention number has decreased as a consequence of the pandemic outbreak.

PSC inspection has covered controlling the regulatory elements such as the certificate and documents, general condition of the navigation equipment, compliance of International Safety Management Code, accommodation and living conditions, crew ability to safe operation, seaworthiness, and machinery areas. Although those elements have been the main indicators of inspection results, differences in PSC regimes, and professional judgments have also impacted the number of deficiencies and detention after the inspection.

In the foregoing circumstances, it can be concluded that the human factor is one of the most important characteristics of PSC. Thus human-related events worldwide have also impacted PSC as well as the other shipboard operations such as crew changes, cargo surveys, maintenance of the vessel, and equipment. On 11 March 2020, World Health Organization (WHO) director declared that COVID-19 can be categorized as Pandemic and spread to more than 114 countries [33]. After the pandemic outbreak was defined by WHO and hit by countries, Flag States decided to postpone all surveys, crew changes, and inspections for several months to protect either ship crew and folk of destination ports. In addition, ports did not allow crew changes, and COVID-19 outbreaks became a major issue for seafarers since around 1.2 million seafarers worked for about 50,000 merchant ships [10] as well.

European Union has also shut down external borders and controlled internal borders to provide practical contamination [15]. However, shipborne trade remained the same, and ships continued to trade within international and European territorial waters. The first response against Novel Coronavirus (2019-nCoV) came from IMO on 31 January 2020 with Circular Letter No.4204 as an acknowledgement of the outbreak based on the World Health Organization (WHO). Followingly, on 28 May 2020, International Maritime Organization (IMO) had issued a Circular Letter No.4204/Add.4/Rev.1 as acknowledgement for the protection of the health of seafarers, when seafarers were unable to repatriate and breach of Maritime Labor Convention (MLC) occurs. Then, the guideline issued 10 of July 2020 No.4204/Add.19/Rev.1 for flag states to guide ship surveys under pandemic outbreak. The circular recalls that the Port State Control (safety net) has been temporarily suspended by some countries, and flexibility of extensions have been granted by some flag States. These two circular letters could be seen as the motives of this paper by implementation means of IMO’s reactions to provide safety on board via PSC and inspection within ongoing pandemic.

## Statistics of Paris MOU and Grey analysis during COVID-19 outbreak

This part of the paper introduces the analysis part is twofold. Firstly, a statistical overview of ship inspection and detention ratio was demonstrated to provide a better understanding of the direct effects of the COVID-19 outbreak. Secondly, a Grey analysis is applied to examine if there is any change in Paris MOU detentions. This part introduces the findings obtained from these analyses. Further on, their results are elaborated on in the following section by discussing the current reflections of the COVID-19 on PSC and inspections.

### Exploring the impacts of pandemic outbreak on Paris MOU according to number of inspection

This section compares ship inspection and detention numbers and all inspection figures for Paris MOU published online by the website. Global trade is based on the shipping industry (90%); however, the COVID-19 pandemic outbreak will affect international trade that affects maritime transport directly [26]. In the short-term period, fewer ships are expected to visit international ports; that’s why fewer inspections are expected to be done compared to previous years. These figures indicate that in March 2020, there had been a dramatic decrease in the number of inspections and deficiency compared to the last five years figures, shown in Table 1.

**Table 1:** Inspection numbers of 2015–2020 at Paris MOU. Source: https://www.parismou.org

Month/ Year	Number of Detentions	Number of Ships inspected	% change in ships inspected	Ships inspected – detention graphics by year
Jan/15	59	1600	–	
Feb/15	54	1389	− 13%	
Mar/15	56	1662	20%	
Apr/15	37	1436	−14%	
May/15	52	1500	4%	
Jun/15	39	1629	9%	
Jan/16	54	1557	–	
Feb/16	43	1446	−7%	
Mar/16	70	1468	2%	
Apr/16	50	1468	0%	
May/16	52	1498	2%	
Jun/16	49	1518	1%	
Jan/17	74	1608	–	
Feb/17	64	1330	−17%	
Mar/17	74	1561	17%	
Apr/17	55	1488	−5%	
May/17	64	1653	11%	
Jun/17	50	1499	−9%	
Jan/18	74	1601	–	
Feb/18	53	1302	−19%	
Mar/18	55	1479	14%	
Apr/18	45	1498	1%	
May/18	57	1641	10%	
Jun/18	38	1573	−4%	
Jan/19	59	1602	–	
Feb/19	48	1385	−14%	
Mar/19	52	1516	9%	
Apr/19	29	1482	−2%	
May/19	45	1665	12%	
Jun/19	38	1497	−10%	
Jan/20	35	1577	–	
Feb/20	40	1345	−15%	
Mar/20	18	746	−45%	
Apr/20	4	244	− 67%	
May/20	14	375	54%	

Table 1 shows five (5) categories; month/year, detention, ships inspected, %change in ships inspected, ships inspected-detention graphics by year. These categories lead us to evaluate key figures and results of inspections for the selected period. Table 1 presents the number of detentions in the European Union from January 2015 to May 2020 within the total number of inspected ships. %Change in ships inspected indicates the percentage of change in the number of inspected ships compared to the previous month of the same year. Ships inspected - detention graphics by year provides a visual comprehension of the changes.

Average inspection numbers for the first six months between 2015 and 2019 is 1493–1536. On the other hand, there is a dramatic decrease in the number of inspections in April 2020 that are affected by the current pandemic outbreak. Table 1. presents that between the years 2017–2019 (last 3 years without COVID - 19), the number of ships inspected is comparable to each other; therefore, Entropy-based Grey Relational Analysis compares these years.

Moreover, the last five years of ship inspection and detention figures indicate that there is a monthly fluctuation in inspection numbers and detained vessels. During all these years, the number of inspections has increased between February and March except for the year 2020. Furthermore, the number of inspections has continued to decrease dramatically in March and April 2020 due to COVID - 19. In addition, the 2015–2019 comparison indicates that the number of inspection and detention has steady figures. After 2020, as a consequence of the pandemic outbreak, port state control officers failed to attend the same number of ships as before. The following section presents in-depth information related to Port State Control and detentions.

### Understanding the trend in ship deficiency-detention factors after COVID-19

A decrease in the number of ships inspection and detention indeed is an expected impact of the COVID-19 pandemic outbreak. Therefore, this section utilizes the entropy-based Grey Relational Analysis to figure out changes in the trend of deficiencies and detentions, analyzing the deficiency codes that PSC uses during the inspection. Grey Relational Analysis (GRA) method can be used as a decision-making method for solving problems involving more than one factor and response within the framework of the relationship level between the analyzed factors and the relationship between changes [15]. In addition, the fact that GRA enables effective analysis with a small number of uncertain data and it is easy to understand has provided some advantages against statistical methods (Wu, [34]; Kung et al., [14]). However, due to inconsistent dimensions or data types in the data to be used in the data analysis process, an approach that can be used in engineering and technical fields is introduced by integrating with the entropy weighting approach on traditional GRA.

To apply the Grey Relational Analysis, the deficiency codes are needed to be determined in order to define the factors of the model. Firstly, these codes are introduced in Table 2, and then the analysis is conducted to present change in Port State Control Officer professional judgement. For this reason, Paris MOU has rose deficiency codes that categorize deficiencies to assist PSCO and policymakers. These codes are also publicly issued on the Paris MOU website.

**Table 2:** Main titles of deficiency codes of Paris MOU. Source: https://www.parismou.org

Deficiency Code	Defective Item (only main title)
01	Certificates & Documentation
02	Structural condition
03	Water/Weathertight condition
04	Emergency Systems
05	Radio communication
06	Cargo operations including equipment
07	Fire safety
08	Alarms
09	Working and Living Conditions
10	Safety of Navigation
11	Lifesaving appliances
12	Dangerous Goods
13	Propulsion and auxiliary machinery
14	Pollution Prevention
15	ISM
16	ISPS
18	MLC, 2006
99	Other

**Table 3:**
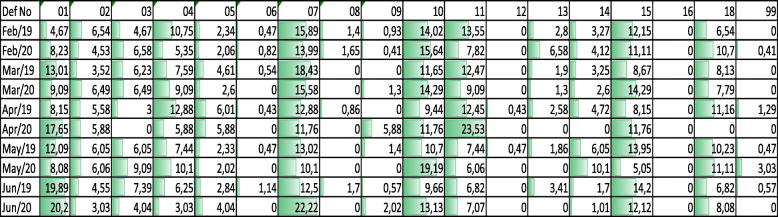
Percentage values of detention reasons by deficiency titles. Source: https://www.parismou.org

Deficiencies noted under the Port state control report have five digits that define the nature of the deficiency, and the first two numbers are the main title. For instance, a deficiency rose from a problem on navigation charts is defined as deficiency code **10**111 – Safety of navigation – Charts. Further data collection has been done by revealing deficiency codes that cause detention between February–June 2019 and February – June 2020.

Percentage values of 2019 and 2020 indicate that in both years, the majority of the detention was caused by fire safety, the safety of navigation, life-saving appliances, ISM Code,^4^ and MLC^5^ 2006 related deficiencies (Table 3). Between February 2019–2020, April 2019–2020, and June 2019–2020 certificate related deficiencies increased.

During the pandemic outbreak, no detention was caused by “06 - cargo operations including equipment”. On the other hand, during the outbreak, fire safety-related deficiencies have decreased except for June 2020 compared to 2019. The detention caused by “13 – propulsion and auxiliary machinery” has decreased compared to 2019 during the pandemic outbreak, and no vessel detained for “16 – ISPS” reason. On the contrary, “10 – the safety of navigation” related deficiencies have risen continuously in the same period, and “01 - Certificate and Documents” related deficiencies have risen except for March 2020.

“99 – Other” deficiency category had a 3,03% detention rate in May 2020. Unfortunately, this category has not been classified, and there is a lack of information on the root cause of this increase. Consequently, a pandemic outbreak and the same period in 2019 had minor changes in detention profile and correlation by month for years 2019 and 2020 is indicated with Table 4.

**Table 4:** Correlation of detention remarks of 2019–2020 from February to June, monthly basis. Source: Authors own calculation based on information (https://www.parismou.org)

Month/ year	Correlation
02/19–20	87%
03/19–20	91%
04/19–20	66%
05/19–20	76%
06/19–20	91%

The correlation has been calculated between February 2019–2020, March 2019–2020, April 2019–2002, May 2019–2020, and June 2019–2020. January has not been calculated because the outbreak started in late February – the beginning of March 2020. It is obvious that there is a strong correlation between 2019 and 2020; even the number of inspected and detained ships decreases dramatically during the pandemic outbreak.

The entropy weighting method objectively reflects the true importance of each factor in the system when determining weights for various factors. The steps of entropy-based grey relevance analysis have been presented in Table 5.

**Table 5:**
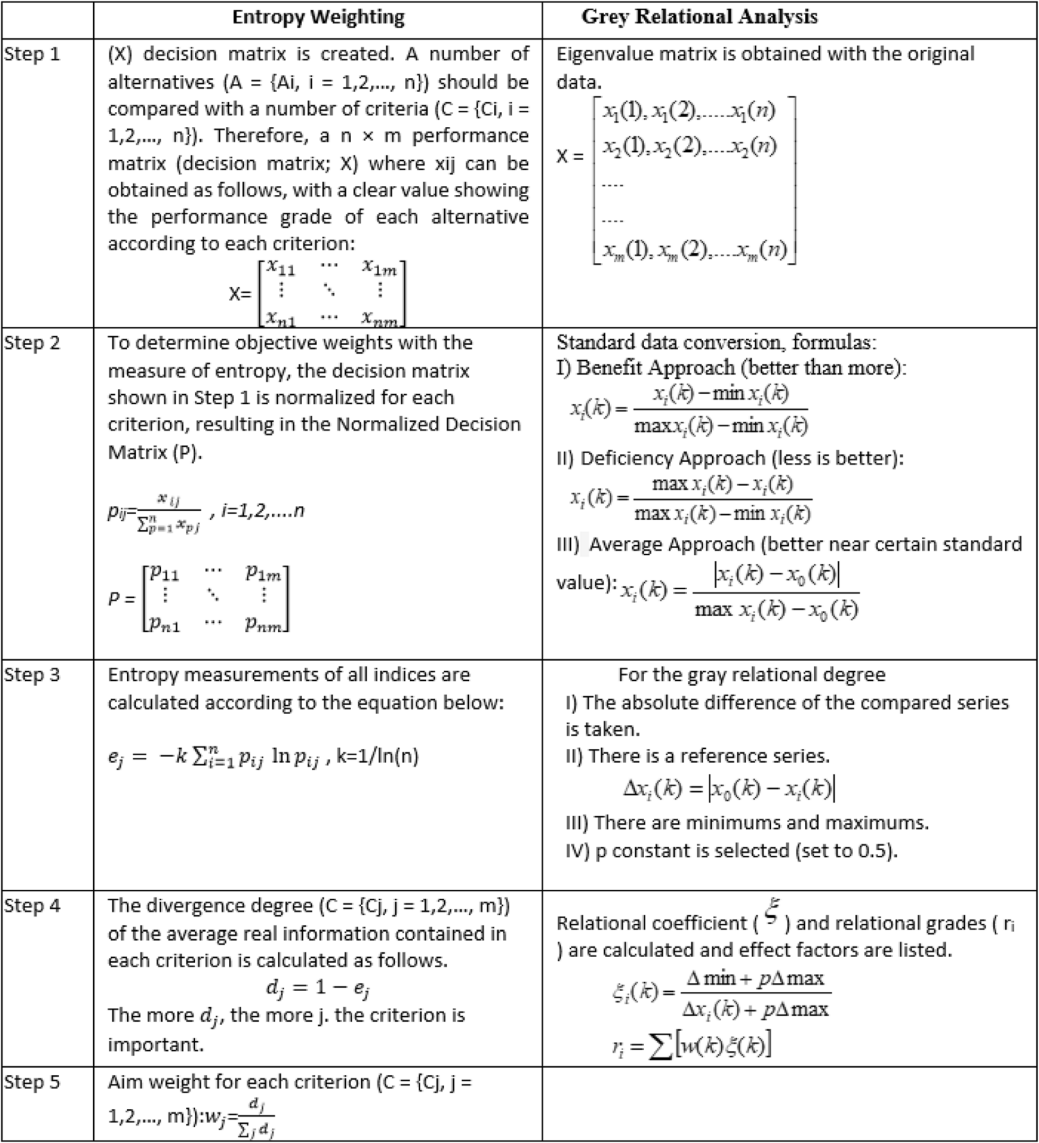
Entropy-Based Grey Relational Analysis (Vatansever and Akgul, [31]; Guzel and Bolat, [7]).

Entropy-based grey relational analysis has also been used for the detention data of Tokyo MOU for the Asian-Pacific Region [15], and it is decided to be an effective rule for understanding the trend in ship deficiency and detention factor. After evaluating the Paris MOU database, 15 factors have been identified in Table 2 as deficiency and detention factors that have records for the 2017–2020 period. To be able to compare the data with the COVID-19 period, data has been assessed for six months of each year. The most predominant factors that have been used for analysis demonstrated in Table 6.

**Table 6:** Ship Deficiency/Detention Factors for Entropy-Based GRA. Source: Determined factors by the Authors based on the information (https://www.parismou.org)

Deficiency/Detention Factors	Content
F1	ISM
F2	Fire doors/opening in the fire
F3	Nautical publications
F4	Charts
F5	Voyage passage plan
F6	Oil record book
F7	Light, shapes, sound-signals
F8	Cleanness of engine room
F9	Auxiliary engine
F10	Seafarers employment
F11	Propulsion main engine
F12	Onboard training
F13	Emergency
F14	Fire detection and alarm
F15	Firefighting equipment

The decision matrix was obtained by the deficiency and detention numbers based on the Paris MOU database between 2017 and 2020. The implementation of the entropy weighting approach has been conducted through the steps of Table 5. Normalization transformation and entropy weightings of nonconformity factors were calculated. Then Grey Relational Correlations have been generated.

When the factors have been ranked for 2017–2020 with a general perspective according to Grey Relational Correlations as in Table 7, it is seen that ranking of the deficiency factors has not a significant change for COVID-19 Pandemic Outbreak. The related data has been given in Additional file 1.

**Table 7:**
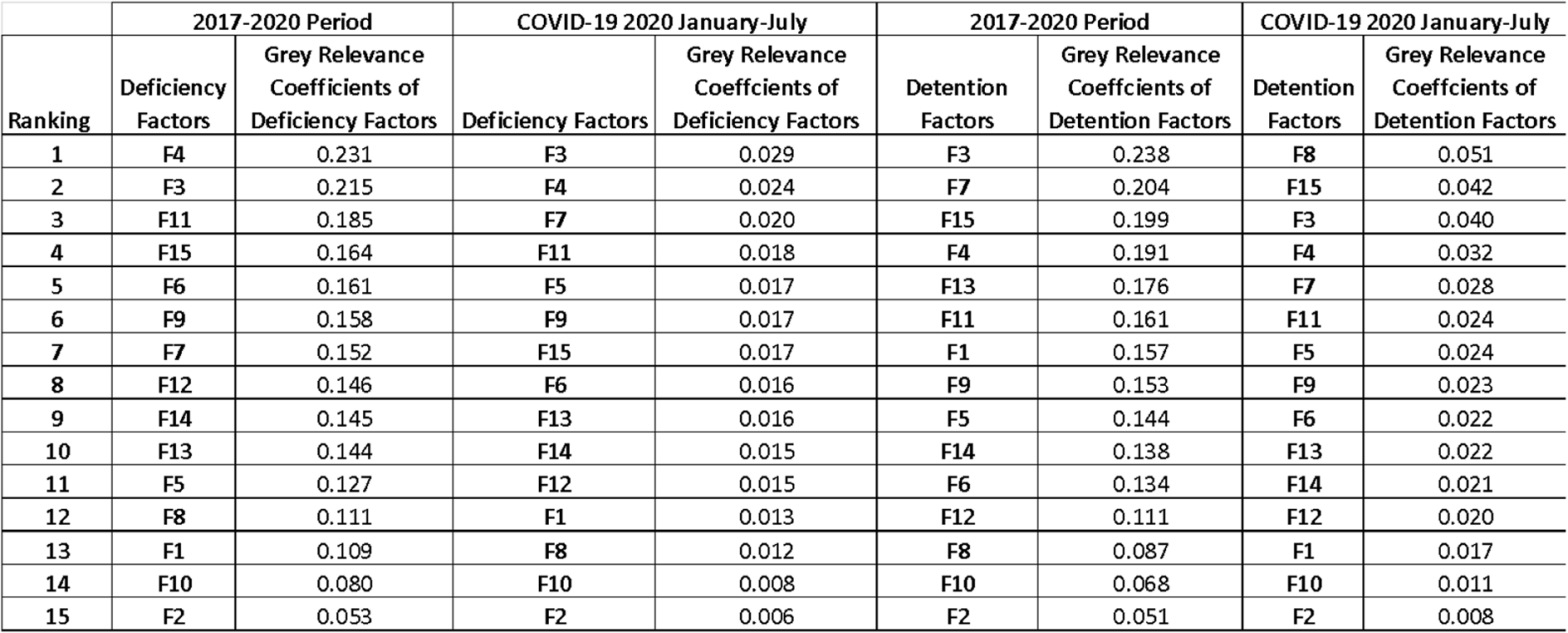
Comparative Ranking for Grey Relational Coefficients. Source: Authors own calculation based on information (https://www.parismou.org).

## Results and discussion

This section of the paper discusses the results of the applied studies above from recent statistics to change the trend of deficiency and detention remarks. The statistical analysis gives an overall understanding from a broader perspective revealing the general consequences of COVID-19 on PSC and decision-making on detentions with Entropy-based Grey Relational Analysis.

After the COVID-19 pandemic outbreak strikes, countries, flag states, ship owners & managers, and crew faced several difficulties with international operations, flights, and travel restrictions that restricted inspections, crew changes, and many more shipboard operations. Later on, IMO has issued a Circular Letter No.4204/Add.19 on the extension of validity of certificates, crew or ship managers likely demonstrate a lack of awareness against expired documents because ship inspection and crew change become a risk for all parties. Then Paris MOU issued guidance related to the impact of COVID - 19 on 26 March 2020. This guidance informed all parties that *‘Whether an inspection takes place remains the decision of the port State.’* [21, 22]*.*

However, during COVID - 19 pandemic outbreak, there is an increase of documents related to detentions during the outbreak, most likely caused by expired ship certificates and crew documents. Increased number of detentions by “10 – Safety of navigation” is likely related to pilot complaints. It is a common practice that in pilot complaints of a vessel to the Port State Control brings PSC officers onboard [24]), so pilots may have a leading part in this detention item during the outbreak.

The decision of the Port State Control Officer done under pressure and limited timing would cause unexpected detentions and unprofessional judgement, so, expected results of this study would demonstrate a lack of inspection for vessels that resulted in high deviation on detention remarks.

However, the monthly correlation is a sign of the effectiveness of the inspections. Percentage values and monthly correlation indicated in Paris MOU statistics present that even under the pandemic outbreak, PSC offices of Europe were working effectively as much as they could. In other words, PSC offices have made a great effort to act as a keeper of maritime standards. Even though the inspected number of vessels decreased dramatically, detained vessels had similar deficiencies compared to last year’s deficiencies. That means the statistical sample unit of vessel selection criteria under the new inspection regime continued to eliminate substandard vessels in European Union territorial waters.

For some aspects, the selection criteria of vessels by port state control are based on statistics and quantitative methods under the “new inspection regime. All ships visiting European countries, MOU inspect vessels under a “selection scheme.” This scheme is determined by a calculation of the history of inspection and factors like vessel age, type, flag, etc. [27]*.*” In this way, authorities and PSCO do not lose unnecessary time to control all vessels at European ports. Not only the time, but an effective ship selection criteria for inspection by Port State Control also saves budget [13]. For this reason, the correlation between the years 2019 and 2020 indicates that PSC Officers have been inspecting vessels without compromising.

The major critics may rise on the crew side that whether the Port State Control Officer has put pressure on the crew by visiting the vessel. Since the current COVID - 19 Pandemic has spread by air, even talking may cause disturbances on the crew who even stayed more than their employment contract duration. Also, decision making on detention by PSCO may be affected under social and time pressure. Significantly, entropy-based grey analysis indicates that there is a significant change in the port state control inspector decision making during the pandemic outbreak.

Entropy-based Grey Relational Analysis of deficiency and detention data for the 2017–2020 period based on Paris MOU records has allowed comparing ship deficiency trends and detention factors for the related period.

**Fig. 1:**
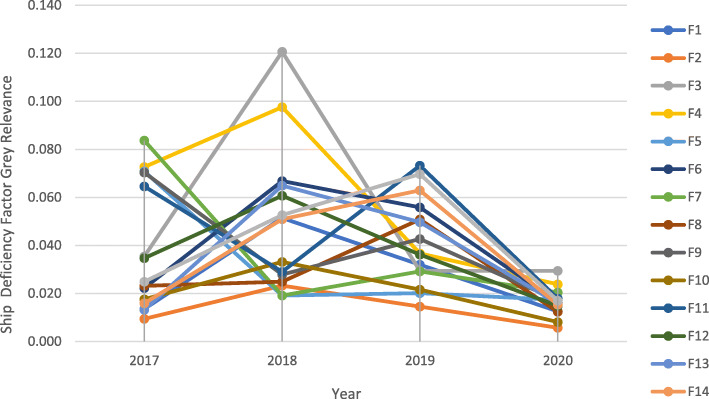
Grey Relevance Correlations of Ship Deficiency Factors between 2017 and 2020. Source: Authors own calculation based on information (https://www.parismou.org)

Grey Relevance Coefficients of ship deficiency factors, shown in Fig. 1, have introduced that F3 (Nautical Publications), F4 (Charts), and F5 (Voyage Passage Plan) has shown slight decrease while the other factors tend to significant declines during the COVID-19 period between January–July 2020 as it decreased in 2019. This decrease can be a fact of Paris MOU policies and taken precautions of shipping lines that have voyages in European Union Waters.

**Fig. 2:**
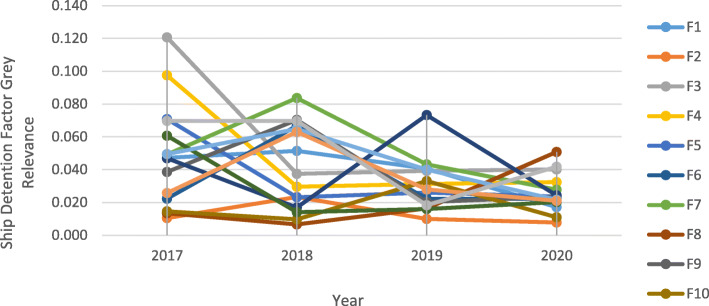
Grey Relevance Correlations of Ship Detention Factors between 2017 and 2020. Source: Authors own calculation based on information (https://www.parismou.org)

When ship detention factors relevance coefficients are evaluated, presented in Fig. 2, it is seen that F11 (Propulsion Main Engine) has a unique decline during the pandemic outbreak. F3 (Nautical Publications), F7 (Light, Shapes, Sound-Signals), F13 (Emergency), F10 (Seafarers Employment) have also continued to a downtrend as in the previous year. F3 (Nautical Publications) and F8(Cleanliness of Engine Room) have shown an increasing trend, although they have a tendency to decrease between 2018 and 2019.

**Fig. 3:**
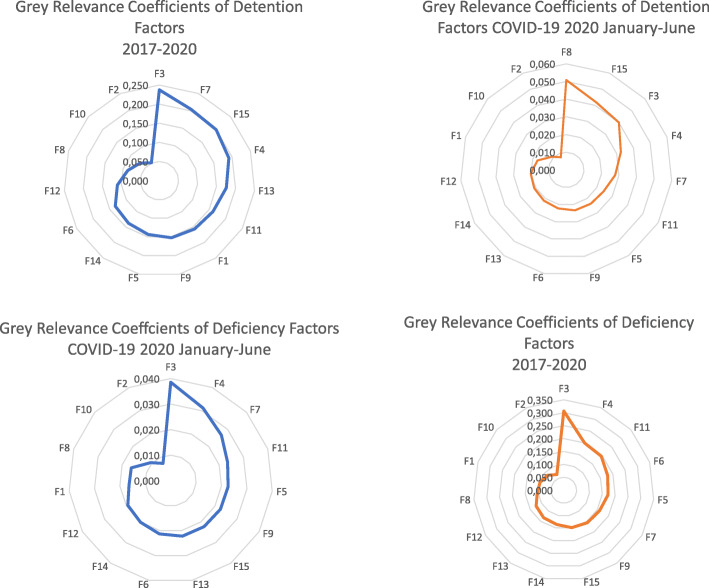
Grey Relevance Coefficients for Deficiency and Detention Factors during 2017–2020 and COVID-19 Pandemic Outbreak. Source: Authors own calculation based on information (https://www.parismou.org)

Consequently, Fig. 3 reveals that the ranking of detention actors has been changed. For the overall relevance, the correlation has shown that F3(Nautical Publications) is the most predominant factor for detention, F8 (Cleanliness of Engine Room) has been ranked as a first factor.

Port State Control Officers inspect the vessel, starting from the documentation. Then proceeds with a physical inspection of cargo, machinery, and accommodation areas. However, the maritime industry is also under the effect of a technological revolution. Currently, certificates are getting digital and verifiable by the internet with *Unique Tracking Code* based on IMO circular FAL.5/Circ.39/Rev.2. Moreover, in practice, some regulations, such as MARPOL Annex VI, SOLAS Regulation I/10(v), may be met with remotely operating devices. It is evident that advanced drone technologies assist in measuring the emission of the vessels [35]. Underwater remote operating vehicles are also an essential tool to inspect ships’ bottom [8]. Enclosed spaces such as cargo tanks and ballast tanks also threaten human life onboard [17]. Under emerging technologies, confined space inspections are also done by remote operating vehicles, and this inspection may also cover thickness and oxygen measurement as well [5].

It is obvious that currently, the majority of the Port State Control inspection has to be done based on the inspector’s presence, and this remains for the near future. On the other hand, developing technology indeed introduces autonomous ships for the maritime industry [28]. Definition of current maritime regulations to be altered by cyber-attacks, intelligent navigation systems, but need for Port State Control to remain the same. Currently, it is necessary to discuss remote Port State Control and inspection means of unmanned autonomous ships against environmental pollution. Since Port State Control is complex and inspects the vessel from top to bottom, current technology is indeed unable to cover an entire inspection. However, there is a potential to reduce the inspection time of PSCO and provide less interaction with the crew.

Currently, the pandemic outbreak will have a long term economic, social, and environmental effects on our lives [4]. There will be inevitable effects on the shipping industry.

## Conclusion

Over the years, regulations made by IMO and implementation practices by Port State Controls, the industry has made a great advancement in maritime safety and environmental protection. These regulations were initiated by marine disasters such as the Titanic, Amoco Cadiz, Exxon Valdez, and many more. This might lead us to reshape existing regulations after the COVID-19 incident in Diamond Princesses.

This paper contributes to the current state of knowledge in the maritime industry by discussing the possible effects of the current pandemic outbreak. Practically, the results of this paper will contribute to the knowledge of ship owners/managers and crew. By demonstrating changes in trends and statistical overview will inform crew and ship owners/managers to act more proactive against deficiencies on ships in terms of being more prepared. Furthermore, this paper will provide a better understanding of PSCO’s and policymakers’ focus under the COVID-19 pandemic outbreak.

The statistics of the Paris MOU present clear evidence that the number of inspections decreased dramatically when COVID-19 hit the European Union. The statistics also indicate that the PSC still works efficiently based on ships inspected - detention ratio. Secondly, Entropy-based Grey Analysis elaborates there is a change in deficiencies and detention judgement of Port State Control Officers. The key highlights of the paper indicate that Paris MOU still works efficiently, but ships are detained even though the Paris MOU secretary issues a guideline on the extension of validity certificates, surveys, crew changes, and services. In this way, this paper gives an overview of changes in the professional judgement of Port State Control Officers; also, Port State Control under Paris MOU has been carried out by either statistical and professional judgement.

Further analysis with Entropy-based Grey Relational Analysis, Port State Control detention remarks indicates that even if a pandemic outbreak affects the number of ships inspected, inspection quality remains the same. It is also a sign for the statistical selection of ships for inspection, and the current inspection regime presents a healthy sample group for inspection.

Overall, current facts indicate that port state control inspection and ‘new inspection regime’ for Paris MOU worked efficiently under the pandemic outbreak. However, deficiency and detention profile had alterations during the outbreak. Since all inspections are carried out by the in-person presence of the Port State Control Officer, the human factor may cause this change. The in-person presence is a treat for possible infection either for the ship crew and the Port State Control Officer since COVID-19 spreads from human to human. This threat may be reduced by application of technological development on inspection methods such as remote inspections by media devices, use of remotely operated vehicle (ROV) for underwater inspection and tank inspections with drones.

The World has been experiencing a new challenge with a pandemic outbreak, COVID-19. While the number of infections increases in Europe, the production chain, agriculture, trade, and tourism had negative effects, and the impact will continue [2]. So, data from February 2020 to June 2020 is only the initial effects of the pandemic outbreak. Further future studies shall analyze wider data when the inspection number reaches the usual trend as in previous years.

There are a total of nine MOUs and the United States Coast Guard in the World, and this article presents insights limited to Paris MOU. Consequently, the Paris MOU is a pioneer memorandum of understanding amongst other institutions that work with statistics in the World. So, the expected long-term result of the outbreak will increase the number of substandard ships all over the World. Comparing ship inspection and detention rates with other MOUs on further studies may provide a broader perspective on the real effects of COVID-19 on maritime transportation. On the other hand, technological development brings advanced solutions for ship inspection. Drone and Remote Operated Vehicles have a great potential to reduce Port State Control Officers’ duration of stay onboard and reduce crew interaction. It shall be predicted by policymakers and inspection mechanisms that the current outbreak shall not cause frailty in the maritime industry by extending the scope and method of ship inspections.

## Supplementary Information

**Additional file 1.**

## Data Availability

The data sets used and/or analysed during the current study, as also shown in the Tables, are available from the public Paris MOU website.

## Footnotes

1 Diamond Princess, British-registered cruise ship, has been widely known by two notable outbreaks of infectious disease. First outbreak happened in 2016 because of norovirus, and the second happened because of SARS-CoV-2 in 2020.

2 Paris MOU: Paris MOU was introduced with the Hague Memorandum on Port State Control and came in force in 1978 aiming to provide better living and working conditions according to ILO Convention No. 147. Shortly after, Amoco Cadiz grounded and European Countries decided to enhance the scope of the memorandum. A Memorandum of Understanding on Port State Control was signed in Paris in January 1982 with 14 European Countries. Currently, there are 27 member countries, and the recent memorandum aims to implement international regulations (safety, security, environmental standards, and adequate living and working conditions).

3 These international conventions are Convention for the Safety of Life at Sea (SOLAS), International Convention for the Prevention of Pollution from Ships (MARPOL), International Convention on Load Lines (LOADLINES), International Convention on Standards of Training, Certification, and Watchkeeping for Seafarers (STCW), Convention on the International Regulations for Preventing Collisions at Sea (COLREG), International Convention on Tonnage Measurement of Ships (TONNAGE), Maritime Labour Convention (MLC) which can also be evaluated under the four pillars of the International Maritime Organization (IMO).

4 ISM Code: International Safety Management Code (ISM Code) aims to provide standards for safe operation and management for shore based and ship based operations in accordance with IMO goals.

5 MLC: Maritime Labor Convention 2006 (MLC) aims to determine standards for seafarer’s rights on working conditions set by International Labor Organization (ILO).
