# Supplementary material for: Port state control at European Union under pandemic outbreak
Source: Eur Transp Res Rev. 2020 Dec 20;12(1):66. doi: 10.1186/s12544-020-00460-4 (PMC7750010; doi:10.1186/s12544-020-00460-4)

**Additional file 1:**

**ENTROPY BASED GREY RELATIONAL ANALYSIS, TABLES OF DEFICIENCIES FOR 2017-2020 Period.** Source: Authors own calculation based on information (https://www.parismou.org)


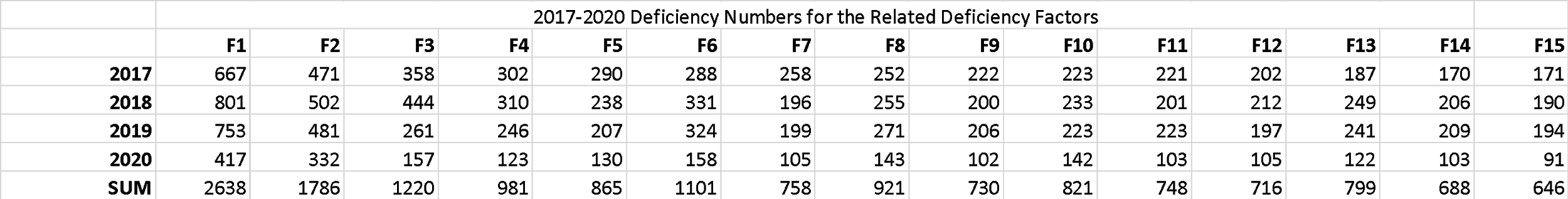


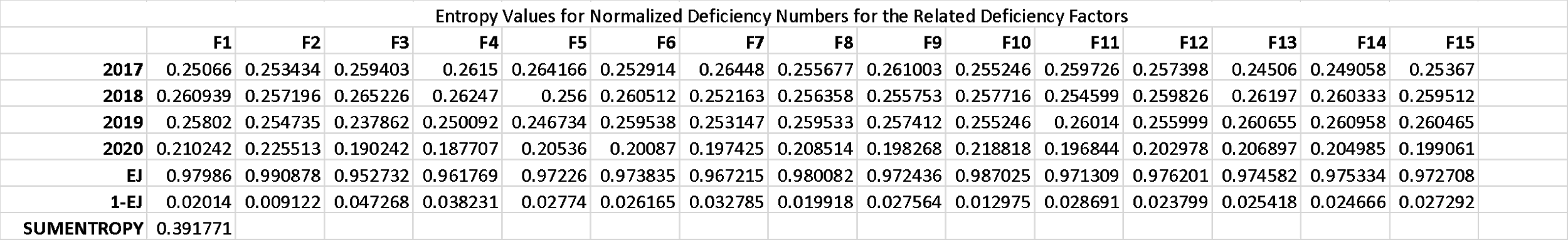

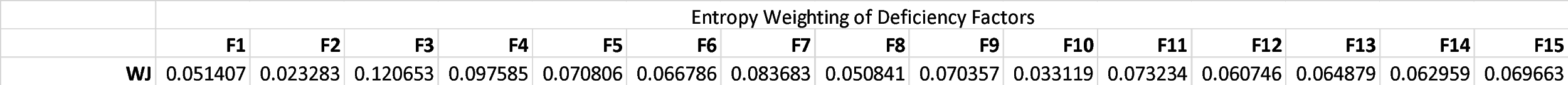


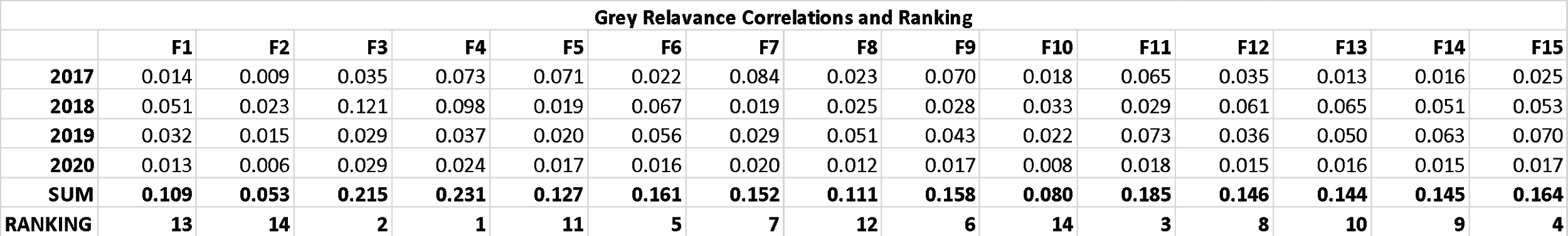


**ENTROPY BASED GREY RELATIONAL ANALYSIS, TABLES OF DETENTIONS FOR 2017-2020 Period.** Source: Authors own calculation based on information (https://www.parismou.org)


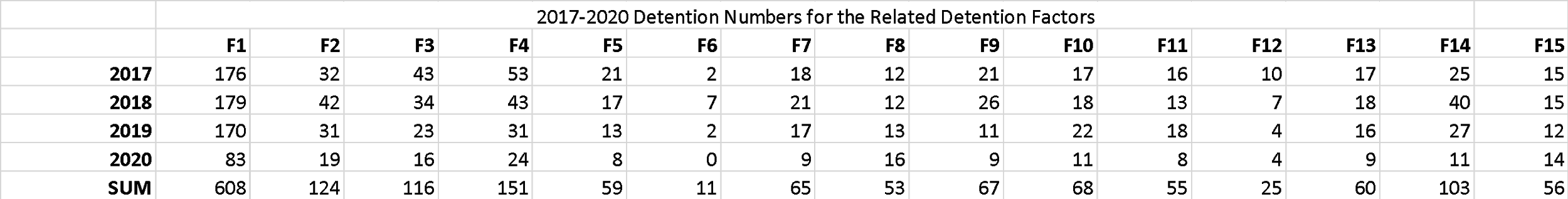


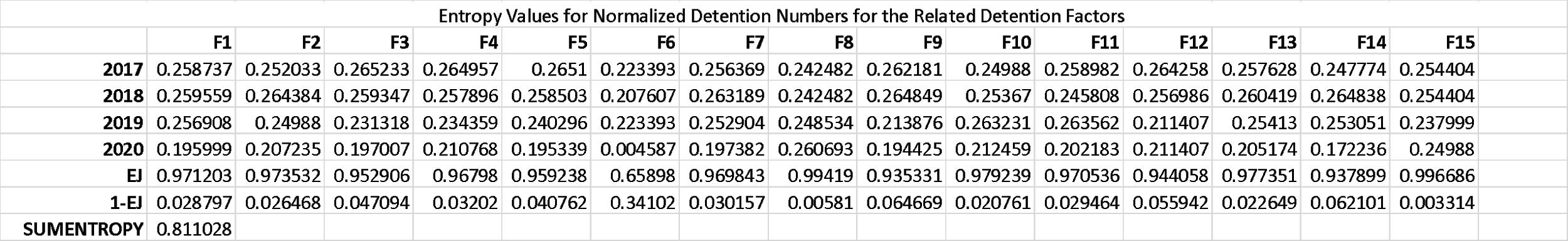


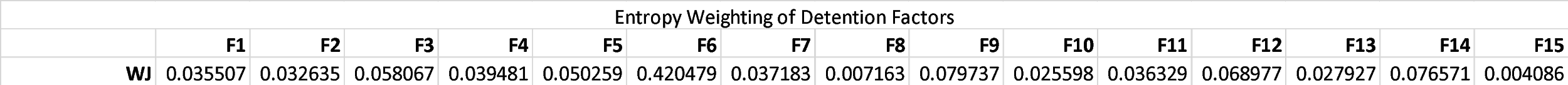

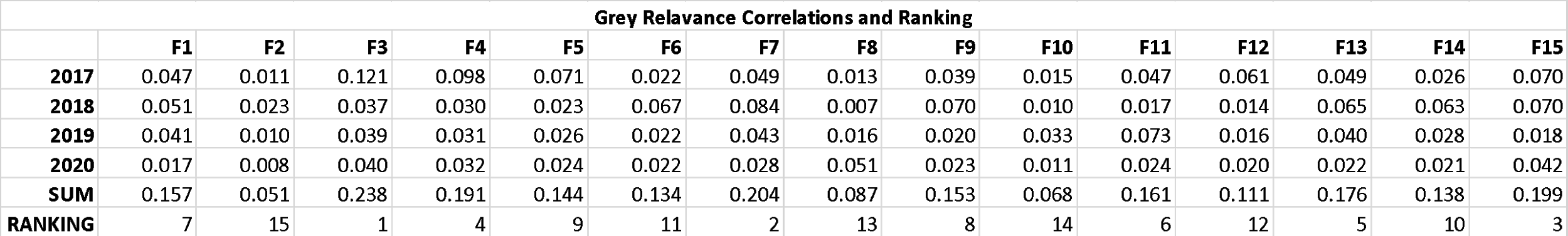

Supplement: Supplementary file 1 — Additional file 1. [file 12544_2020_460_MOESM1_ESM.docx]
